# Resolutions of Respect—Dr. W. H. Dwinelle

**Published:** 1896-05

**Authors:** 


### Obituary.

### RESOLUTIONS OF RESPECT—DR. W. H. DWINELLE.

THE rapid years have gathered one more of the great men of our profession to his final rest.

Dr. Wm. H. Dwinelle, whose life we commemorate, and whose death we mourn, was one of the great figures in the early days of our young profession.

He was born in Cazenovia, N. Y., where he died at the homestead on February 13, 1896, seventy-six years of age.

Entering our profession at a time when it was struggling for recognition among the learned professions, he brought to it the influence of a remarkable personality, and through his varied attainments, and by his energy and hopeful confidence he helped, as few others did, to place it upon a secure foundation among the learned and liberal professions of the world.

Fitted for the practice of medicine and surgery, he yet saw in the specialty of dentistry a wider field for the exercise of his peculiar genius, and he entered upon his work with boundless enthusiasm.

This is shown by his numerous inventions, his brilliant operations, and his contributions to the professional literature of his time. It is also warmly attested by the few surviving companions of those early days.

He assisted in the formation of the first dental college, and was instrumental in establishing the *American Journal of Dental Science*,—one of the most dignified and influential journals our profession has produced.

He performed surgical operations in the oral cavity that were the admiration of the general surgeons of the day, and he performed operations upon the teeth that had never been attempted before.

Many examples of his work are still in existence, to testify to his remarkable ingenuity, and to his unusual skill.

A man of warm heart and generous impulses, he freely gave to all who came; his office was always open, and he was ever ready to show his instruments and his methods to any one who desired to learn.

Having practised medicine and surgery before he entered the dental profession, he commanded the confidence of physicians and surgeons, and was thereby able to help, in an unusual degree, to secure recognition for our specialty, and he stood for many years as a bond between the parent profession and its young offspring.

A man of literary tastes, and a devoted lover of art in all its forms, he was able to reflect credit upon our profession at a time when such influences where more needed than at present.

A man of tender sensibilities, he was a genial companion, and his wide sympathies and varied talents made him a great favorite among cultured people.

He was a man of so many gifts that he could have been a poet, an actor, an artist, a sculptor, or a *littérateur*, this wide range of talent made him always an agreeable friend.

Before the bar he would have been a great advocate; in the medical profession he would have been a great physician or a great surgeon. He chose to be a great dentist.

For this we honor his memory, and we think it fitting that this society, once presided over by him, should place on record its appreciation of him while living, and its sorrow for his death.

A. R. Starr,
Wm. Jarvie,
S. G. Perry,
*Chairman.*

